# Supplementary material for: Validation of an ultra-short global quality of life scale in a large population-based health survey
Source: PLoS One. 2024 Aug 29;19(8):e0307539. doi: 10.1371/journal.pone.0307539 (PMC11361658; doi:10.1371/journal.pone.0307539)
Supplement: Attachment — Submitted filename: Reviewer Ejoke Ufuoma concerns_PLOS ONE.docx [file pone.0307539.s001.docx]

**Ejoke Ufuoma concerns**

Overall, the article presents a well-structured and clear validation study of the Essential QoL-3 (EQoL-3) scale. The introduction effectively sets the stage for the need for a concise yet robust QoL measure, particularly in mental health and addiction research. The literature review is comprehensive, covering the importance of QoL assessment, limitations of existing measures, and the theoretical framework underlying the EQoL-3.

The methods section is detailed and transparent, providing sufficient information to replicate the study. The sample size is adequate, and the data analysis is appropriate for the research questions.

The results section is clearly presented, with tables and text that effectively convey the findings. The EQoL-3 demonstrates excellent internal consistency, discriminant validity, and convergent validity, supporting its use as a valid and useful measure of QoL.

The discussion section effectively summarizes the main findings, relates them to the broader literature, and discusses the implications of the study. The authors convincingly argue that the EQoL-3 offers a concise and robust measure of QoL that can be used in various contexts.

**However, some minor suggestions for improvement include:**

- Providing more context for the EQoL-3 scale, such as its development process and previous uses
- Including more information about the sample demographics and characteristics
- Discussing potential limitations of the study, such as the reliance on self-report measures
- Exploring future research directions and potential applications of the EQoL-3 in more detail

**To elaborate on the question on whether the statistical analysis been performed appropriately and rigorously?**

I wonder why only a multigroup CFA was conducted. In current practice, several measurement models are tested, for instance a bifactor model, an ESEM and even a bi-factor ESEM. I suggest the authors do a more robust analysis or get the support of a statistician to do this.

Also, while the author/s conducted convergent and discriminant validity, other types of reliability such as test-rest reliability need to be determined for a psychometric study.

The author/s did not acknowledge any assumptions made during their data analysis.

**The following concerns below should be further addressed:**

How did you ensure data quality?

Was the study pre-registered? If not, why not?

In your CFA, which identification strategy did you employ and why (unit variance identification, or unit loading identification)?

Which methods of setting factor loading did you use and why? Fixed factor loading or freely estimated factor loading?

Overall, the article presents a strong validation study of the EQoL-3 scale, and with some minor revisions, it has the potential to make a significant contribution to the field of QoL research.
