# Supplementary material for: Validation of an ultra-short global quality of life scale in a large population-based health survey
Source: PLoS One. 2024 Aug 29;19(8):e0307539. doi: 10.1371/journal.pone.0307539 (PMC11361658; doi:10.1371/journal.pone.0307539)
Supplement: Attachment — Submitted filename: Response to Reviewers.docx [file pone.0307539.s002.docx]

**Response to Reviewers**

**Editorial comment:**

Journal Requirements:

When submitting your revision, we need you to address these additional requirements.

1. Please ensure that your manuscript meets PLOS ONE's style requirements, including those for file naming. The PLOS ONE style templates can be found at 
<https://journals.plos.org/plosone/s/file?id=wjVg/PLOSOne_formatting_sample_main_body.pdf> and 
<https://journals.plos.org/plosone/s/file?id=ba62/PLOSOne_formatting_sample_title_authors_affiliations.pdf>

2. Please review your reference list to ensure that it is complete and correct. If you have cited papers that have been retracted, please include the rationale for doing so in the manuscript text, or remove these references and replace them with relevant current references. Any changes to the reference list should be mentioned in the rebuttal letter that accompanies your revised manuscript. If you need to cite a retracted article, indicate the article’s retracted status in the References list and also include a citation and full reference for the retraction notice.

RESPONSE:

1. We have checked the style requirements and have adjusted the filenames and manuscript to meet the journal style. Changes due to formatting requirements have not been marked with “track changes”
2. We have checked the reference list. We were not able to find retracted manuscripts in the list.

**Reviewer comments:**

**Reviewer #1: Ejoke Ufuoma concerns**

Overall, the article presents a well-structured and clear validation study of the Essential QoL-3 (EQoL-3) scale. The introduction effectively sets the stage for the need for a concise yet robust QoL measure, particularly in mental health and addiction research. The literature review is comprehensive, covering the importance of QoL assessment, limitations of existing measures, and the theoretical framework underlying the EQoL-3.

The methods section is detailed and transparent, providing sufficient information to replicate the study. The sample size is adequate, and the data analysis is appropriate for the research questions.

The results section is clearly presented, with tables and text that effectively convey the findings. The EQoL-3 demonstrates excellent internal consistency, discriminant validity, and convergent validity, supporting its use as a valid and useful measure of QoL.

The discussion section effectively summarizes the main findings, relates them to the broader literature, and discusses the implications of the study. The authors convincingly argue that the EQoL-3 offers a concise and robust measure of QoL that can be used in various contexts.

RESPONSE: Thanks for the encouraging feedback on our manuscript.

However, some **minor suggestions for improvement include:**

• Providing more context for the EQoL-3 scale, such as its development process and previous uses

RESPONSE: There was information about the development process from line 91 in the manuscript. The development of the EQoL-3 scale was based on a review of QoL measures used in European public health surveys undertaken by the Norwegian Institute of Public Health (NIPH) (Nes et al., 2018). Based on the findings, the review recommended that QoL surveys in Norway should include the mentioned three dimensions (the cognitive, affective and eudaimonic dimension) to provide a comprehensive assessment. The recommendations were based on OECD guidelines for measuring subjective well-being. We have now included this sentence and the reference to the OECD guidelines (OECD, 2013). Note that the second paragraph on page 6 includes information about how the EQoL-3 diverges from the NIPH’s original suggestion. Thus, this paragraph describes the authors’ suggestion on how to condense the measure into only three indicators. On line 103 we have described that the measure has been used previously in two articles, but it has not been formally validated before.

• Including more information about the sample demographics and characteristics

RESPONSE: There was a limited number of variables covering sociodemographic characteristics in the survey. We have added data on whether respondents were native Norwegians or immigrants and expanded on the “living arrangement” variable (see description of sample page 14 and Table 2).

• Discussing potential limitations of the study, such as the reliance on self-report measures

RESPONSE: We have added the limitation that test-retest reliability was not examined, as recommended by the reviewer, see comment down below. Regarding reliance on self-report measures, we do not think of it as a limitation, as subjective QoL can solely be assessed through self-reports.

• Exploring future research directions and potential applications of the EQoL-3 in more detail

RESPONSE: We have elaborated more on the application of the EQoL-3 in epidemiological and clinical research in the “Methodological consideration section”

To elaborate on the question on whether the statistical analysis been performed appropriately and rigorously?

I wonder why only a multigroup CFA was conducted. In current practice, several measurement models are tested, for instance a bifactor model, an ESEM and even a bi-factor ESEM. I suggest the authors do a more robust analysis or get the support of a statistician to do this.

RESPONSE: In latent analyses, the model fit statistics are used to verify whether data fits the hypothesized models, and as such, they speak to the robustness of the models. Our result section lists the standard cut-off for model fit statistics and all fit statistics were acceptable or good, as described in the result section. The procedures for the CFA and latent regression analyses were undertaken as described in standard SEM textbooks and articles (Brown, 2015; Byrne et al., 1989; Kline, 2016). These are referred to in the statistical method section. We are familiar with these techniques from previous studies and previous guidance from a statistician (Vederhus et al., 2016; Vederhus et al., 2019).

The multigroup CFA was used for the discriminant analysis, i.e., whether the EQoL-3 was perceived in a similar way across groups. If these analyses are not undertaken, means differences can be due to measurement error. In this case, scalar equivalence was achieved, which is the desirable level of equivalence, and latent mean differences could be reported (Byrne et al., 1989). Thus, the invariance procedures and the confirmation of equivalence of the measure across groups also speak to the robustness of our results.

We here wanted to examine the EQoL-3 in the most stringent way and evaluated its a priori hypothesized structure with a CFA. Thus, exploratory approaches, such as an exploratory factor analysis (EFA), were not relevant. The indicators of the EQoL-3 were considered to represent QoL as a univariate construct, i.e., as a measure of the essential features of QoL. We have now specified this better in the Methods section (line 141). As the hypothesized model worked well, there was no need to examine other solutions. Regardless, a model with three indicators would not suffice in a bifactor model, as it would be under-identified, i.e., not having enough information to estimate all parameters.

Based on the above elaborations, we have chosen to retain the analysis as it was.

Also, while the author/s conducted convergent and discriminant validity, other types of reliability such as test-rest reliability need to be determined for a psychometric study.

RESPONSE: We acknowledge that it would be ideal to include test-retest reliability. We have now inserted this in the Methodological consideration section as a limitation.

The author/s did not acknowledge any assumptions made during their data analysis.

RESPONSE: We have now noted in the Statistical Analyses section that an important assumption for SEM models was met, i.e., a sufficiently large sample size (line 232). Common requirement for regression models, such as "multicollinearity" is traditionally associated with correlations among formative variables, i.e., a variable constructed as an average score of observed indicators. Thus, in such models it is important to check assumptions, as analyses based on formative variables are handled without measurement errors, an assumption usually violated in practice (Kline, 2016, p. 213). In a SEM framework, these measurement errors are accounted for. We note that our latent regression model was a fully latent model, i.e., all examined variables, QoL, mental distress and perceived mastery, were handled as latent constructs.

When indicators are measured on an ordinal scale with fewer steps (e.g., a 5-point ordinal scale), Maximum Likelihood Estimation with Robust Standard Errors (MLR) is sometimes used. MLR is an estimation method that provides standard errors and chi-square statistics that are robust to non-normality. In this study, the EQoL-3 were scored on 0-10 point scales, so the standard Maximum Likelihood (ML) estimator was sufficient (line 229). Nevertheless, we also examined the models with MLR and found no differences with the ML results. Therefore, we reported the results using the most common and straightforward procedures.

The following concerns below should be further addressed:
How did you ensure data quality?

RESPONSE: As noted in line 120, the data for this investigation were obtained from a national survey conducted by Statistics Norway (SSB) under the commission of the Norwegian Directorate of Health. SSB is a highly reputable agency with extensive experience in conducting national surveys, ensuring high data quality.

Additionally, we conducted a descriptive check of all variables used in this study as part of our standard procedures prior to any statistical analyses. This routine check confirmed the integrity and appropriateness of the data. Given that this step is a standard procedure, we did not detail it in the Statistical Analyses section, but it is implicit in our data handling process.

We also noted that the data set had minimal missing values, with coverage exceeding 99.7% for all variables (line 238). The standard method of handling missing data (Full Information Maximum Likelihood – FIML) was used. FIML is preferred over traditional methods like listwise deletion because it makes use of all available data points. We have now added this to the Statistical analyses section (line 230).

Was the study pre-registered? If not, why not?

RESPONSE: The study was not pre-registered. To our knowledge, there is no requirement to register studies based on cross-sectional data.

In your CFA, which identification strategy did you employ and why (unit variance identification, or unit loading identification)?

RESPONSE: As per standard in SEM, the loading of one indicator of the latent factor was fixed to 1.0 in unstandardized models to set the scale of the latent variables. This basic SEM approach is commonly not detailed in the Statistical analyses section.

Which methods of setting factor loading did you use and why? Fixed factor loading or freely estimated factor loading?

RESPONSE: See response above. In an unstandardized SEM model, a fixed factor loading is used for identification purposes. In a standardized SEM model, the focus shifts from identification to standardizing the estimates of model parameters. Thus, the factor loadings are freely estimated. To allow for identification, the variance of the latent factor is fixed to 1.0. This approach allows for the interpretation of standardized factor loadings, which are reported in the Results section (see line 272). As the above procedures are standard output of Mplus analyses and SEM, they are not commonly detailed in the Statistical Analysis section.

Overall, the article presents a strong validation study of the EQoL-3 scale, and with some minor revisions, it has the potential to make a significant contribution to the field of QoL research.

RESPONSE: Thank you for these positive feedbacks on our manuscript!

**Reviewer #2: Dear authors**

Congratulations on the manuscript! I found it very interesting, especially because it is a simple and quick instrument for assessing QoL, very useful especially for use in large population studies. The inclusion of aspects of cognitive, affective and eudaimonic dimensions are very welcome, as most of the instruments used in research are related to health-related quality of life, which may not have much meaning or importance for individuals without an actual health problem health. The objectives and methodology of the work are also well described. In general, I have no further observations to make, other than to congratulate you once again.

RESPONSE: Thank you for the positive feedback on our manuscript!

**References**

Brown, T. A. (2015). *Confirmatory factor analysis for applied research* (2nd ed.). Guilford Press.

Byrne, B. M., Shavelson, R. J., & Muthen, B. (1989). Testing for the equivalence of factor covariance and mean structures: The issue of partial measurement invariance. *Psychological Bulletin*, *105*(3), 456-466. <https://doi.org/10.1037/0033-2909.105.3.456>

Kline, R. B. (2016). *Principles and practice of structural equation modeling* (4th ed.). Guilford Press.

Nes, R. B., Hansen, T., & Barstad, A. (2018). *Quality of Life. Recommendations for a better way of measuring it. [Livskvalitet. Anbefaling for et bedre målesystem].* . The Norwegian Directorate of Health.

OECD. (2013). *OECD Guidelines on Measuring Subjective Well-being*. OECD Publishing.

Vederhus, J. K., Clausen, T., & Humphreys, K. (2016). Assessing understandings of substance use disorders among Norwegian treatment professionals, patients and the general public. *BMC Health Services Research*, *16*(1), 52. <https://doi.org/10.1186/s12913-016-1306-9>

Vederhus, J. K., Kristensen, O., & Timko, C. (2019). How do psychological characteristics of family members affected by substance use influence quality of life? *Quality of Life Research*, *28*(8), 2161-2170. <https://doi.org/10.1007/s11136-019-02169-x>
